# Supplementary material for: Tumor-Associated and Systemic Autoimmunity in Pre-Clinical Breast Cancer among Post-Menopausal Women
Source: Biomolecules. 2023 Oct 24;13(11):1566. doi: 10.3390/biom13111566 (PMC10669589; doi:10.3390/biom13111566)
Supplement: Supplementary file 1 [file biomolecules-13-01566-s001.zip › biomolecules-2541849-Supplemental materials_24Oct23_clean-updated.pdf]

Supplemental Materials: **Tumor-associated and systemic autoimmunity in pre-clinical breast cancer among post-menopausal women**

Supplemental Table 1. Characteristics of women with and without ANA in the WHI

	ANA-positive N=1511	ANA-negative N=8473	p-value
Age in years (SD)	64.0 (7.3)	63.9 (7.3)	0.55
**Other antibodies, drugs, estrogen replacement**	%	%	
Anti-CCP positive	12	7	<0.0001
Anti-RF positive	26	15	<0.0001
Current DMARD use	7	5	<0.0001
Current Prednisone use	5	3	0.003
Current estrogen use			
E-only	22	23	0.65
E+P	14	12	
**Other Characteristics**	%	%	
Lupus (self-report, baseline or follow-up)	13	6	<0.0001
Prevalent cancer (self-reported) – Any	10	10	0.81
Incident cancer (adjudicated)			
Any Cancer[1]	15	15	0.81
Leukemia/lymphoma	2	1	0.071
Breast cancer[2]	N=85 (5.6%)	N=533 (6.2%)	
ER-positive	89	80	0.074
Invasive	80	84	0.33

[1]Baseline history of any self-reported cancer, except for non-melanoma skin cancer.
[2]Of these, prior history of breast cancer was reported at baseline on 8% of ANA-positive cases, and 12% of ANA-negative cases. Tumor ER status was missing or unknown on 97 ANA negative cases and 15 ANA positive cases.

Supplemental Table 2. Screening Log2TAA ratios comparing values in ANA-positive with ANA-negative women, by case status

	Cases				Controls		
Gene Symbol	p-value[1]	Mean[2]	Median		p-value[1]	Mean[2]	Median
AADAT	0.141	0.88	0.87		0.961	1.00	1.04
ABCA8	0.057	1.63	1.29		0.243	1.18	1.22
ABCA8	0.403	1.13	1.13		0.259	1.15	1.18
ACO1	0.134	1.18	0.99		0.391	0.91	0.92
ACP1	0.174	0.74	0.75		0.732	1.07	1.16
ACTA2	0.556	0.95	0.90		0.733	0.97	0.99
ACTA2	**0.021**	0.84	0.86		0.629	0.96	0.88
ADCK1	0.835	1.07	1.05		0.473	1.20	1.10
ADCK1	0.742	0.91	0.97		0.478	1.20	1.25
AHNAK	0.259	0.89	0.85		0.985	1.00	1.00
ALDOB	0.286	1.33	1.32		0.587	0.88	0.95
ALDOC	0.423	0.94	0.89		0.593	0.95	0.86
ALPL	0.853	1.01	1.01		0.828	1.03	0.90
ALPL	0.549	0.91	0.81		0.606	0.97	1.02
ANGPT1	0.821	1.09	1.24		0.914	1.03	1.32
ANKHD1	0.988	1.01	0.85		0.666	1.13	1.04
ANKRD20A11P	0.497	0.85	1.01		0.130	1.32	1.54
APMAP	0.510	0.94	1.00		0.789	0.98	0.99
AQP1	0.323	1.22	0.97		0.452	1.16	0.95
AQP5	0.198	0.91	0.84		0.581	0.96	0.93
ARD1B/NAA11	0.326	0.84	0.78		0.412	0.87	0.96
ASF1	0.192	0.87	0.81		0.581	0.94	0.97
ATP1B3	0.551	1.06	0.94		0.371	1.10	0.92
ATP5J	0.953	1.01	1.00		0.140	1.16	1.10
B3GNT5	0.859	0.93	0.78		0.775	0.88	1.02
B4GALT7	0.629	0.90	0.99		0.633	0.96	0.93
B4GALT7	0.130	0.88	0.84		0.777	0.97	0.94
BCAP31	0.376	0.60	0.84		0.315	1.72	2.09
BNIP1	0.888	0.94	1.08		0.352	1.46	1.10
BRSK1	0.987	1.00	0.80		0.953	0.99	1.12
BTN2A1	0.792	0.92	1.22		0.767	1.09	1.14
C13orf24	**0.008**	0.78	0.84		0.994	1.00	0.92
C17orf58	0.961	0.99	0.92		0.617	0.96	0.93
C17orf58	0.097	0.88	0.87		0.544	0.95	0.94
C5orf45	**0.032**	0.79	0.79		0.397	0.93	0.81
C9orf16	0.941	1.01	1.03		0.165	1.20	1.10
CA12	0.234	0.76	0.76		0.315	0.81	0.87

CA7	0.627	1.07	1.02	0.878	0.98	1.03
CA9	0.281	0.66	0.88	0.163	0.50	0.85
CA9	0.682	0.82	0.95	0.901	0.96	0.87
CACNA1D	0.395	1.27	1.10	0.329	1.22	1.15
CALM3	0.510	0.94	0.91	0.982	1.00	0.88
CALML5	0.645	0.81	1.00	0.353	1.51	5.54
CARD14	0.172	0.87	0.83	0.924	1.01	1.00
CARD14	0.110	0.84	0.84	0.392	1.25	0.96
CBLB	0.403	1.26	0.87	0.115	1.21	1.34
CCDC33	0.415	0.91	0.83	0.069	0.83	0.89
CCDC33	0.083	0.84	0.82	0.373	0.91	0.88
CDC23	0.286	1.30	1.15	0.478	1.13	1.09
CDC23	0.541	1.11	1.06	0.881	1.04	1.23
CDC25C	0.618	0.94	0.91	0.399	1.22	0.84
CDC25C	0.331	0.91	0.88	0.650	1.05	0.93
CDKN2AIP	0.755	0.96	0.86	0.356	1.13	1.06
CENPM	0.681	1.09	1.14	0.357	1.14	1.15
CHCHD6	0.247	0.87	0.86	0.956	1.01	0.95
CLIP3	0.207	0.92	0.97	0.911	1.01	0.93
CNIH2	0.628	1.14	0.95	0.754	0.96	0.87
COMT	0.720	1.06	1.26	0.490	0.89	0.84
COX15	0.675	1.05	1.08	0.452	1.21	1.16
COX15	0.802	0.94	1.14	0.775	1.03	1.10
COX15	0.543	1.08	1.16	0.377	1.10	1.14
CSNK1D	0.942	0.98	1.00	0.662	1.09	1.29
CSNK1E	0.699	0.88	0.79	0.128	1.68	1.55
CSTF2	0.662	1.16	0.87	0.814	0.95	0.95
CTSB	0.568	0.95	0.91	0.778	1.03	0.89
CXorf56	0.289	0.89	0.87	0.895	0.98	0.90
DECR2	0.611	1.21	1.00	0.017	2.01	1.00
DHX9	0.854	0.98	0.93	0.559	1.07	0.90
DHX9	0.616	0.96	0.91	0.905	0.99	0.96
DHX9	0.134	0.85	0.78	0.306	1.14	1.11
DRAM	0.129	0.91	0.97	0.345	0.93	0.93
DRAM	0.091	0.88	0.89	0.689	0.96	0.92
DUSP26	**0.018**	1.43	1.17	0.492	1.14	1.15
EFCAB2	0.293	0.92	0.92	0.509	0.92	0.95
EIF5A	0.093	1.26	1.07	0.796	0.97	0.83
ENO3	0.203	0.89	0.89	0.580	0.96	0.88
ENO3	0.329	0.93	0.93	0.932	0.99	0.95
FAM119A /METTL21A	0.121	0.86	0.80	0.758	0.97	1.01
FAM19A4	0.413	1.10	1.12	0.436	1.22	1.01

FAM19A4	0.877	0.96	1.13	0.366	1.26	1.22
FAM19A4	0.912	1.02	1.07	0.318	1.13	1.13
FGFR1	0.505	0.89	0.92	0.582	0.93	1.07
FGFRL1	0.224	0.88	0.86	0.971	1.00	0.93
FLII	0.560	1.15	1.14	0.745	1.08	1.23
FLII	0.600	1.14	0.99	0.755	1.08	1.22
GAPDH	0.751	0.96	0.99	0.528	0.93	0.88
GAPDHS	0.769	1.06	0.83	0.303	1.29	0.96
GAPDHS	0.355	0.90	0.94	0.545	1.06	0.96
GNB1L	0.541	1.24	1.29	0.425	1.24	1.08
GNPTG	0.615	1.20	1.08	0.175	1.39	1.60
GPC4	0.617	0.97	0.93	0.230	1.10	1.00
GPX7	0.165	1.20	1.18	0.404	1.12	1.05
Gro-alpha	0.949	1.02	0.82	0.982	0.99	0.80
GTDC1	0.514	0.78	1.06	0.989	1.00	0.95
GTDC1	0.959	0.99	0.92	0.616	0.95	1.01
GTDC1	0.338	0.93	0.91	0.731	1.04	1.14
GTF2I	0.841	0.95	1.06	0.461	1.19	1.11
H2AFV	0.596	0.92	0.98	0.306	0.81	0.82
HABP2	0.451	1.09	1.16	0.448	1.12	1.12
HABP2	0.826	1.04	1.09	0.111	1.22	1.09
HAVCR2	0.104	1.40	1.08	0.825	0.97	0.92
HAVCR2	0.333	0.90	0.86	0.517	1.07	1.00
HIBADH	0.414	0.91	0.89	0.970	1.00	1.02
HNRNPUL1	0.817	0.90	1.00	0.147	0.50	1.00
HNRPA1	0.396	0.90	0.91	0.508	1.09	1.10
HNRPF	0.157	0.88	0.91	0.746	1.03	0.99
HNRPH3	0.273	0.89	0.91	0.352	0.93	0.93
HNRPH3	0.057	0.86	0.88	0.539	0.93	1.07
HNRPK	0.619	0.95	0.88	0.808	1.02	0.84
HSD17B10	0.809	1.07	0.83	0.432	1.20	1.29
HSPA2	0.614	0.84	0.97	0.720	0.89	1.11
HSPB9	0.110	0.86	0.85	0.673	0.96	0.90
HSPB9	0.043	0.87	0.92	0.789	0.97	0.84
IGF1R	0.323	0.66	1.00	0.898	1.08	1.41
IGLV3-25	0.899	1.07	1.25	0.384	1.60	1.21
IL12RB1	0.801	1.02	1.03	0.346	1.24	1.05
IL12RB1	0.216	0.88	0.82	0.909	1.01	1.09
IL3	0.293	1.27	0.99	0.273	0.90	0.92
IL3	0.843	1.06	1.00	0.930	0.98	0.98
IL3	0.963	0.99	0.99	0.615	1.12	0.99
INO80E	0.358	0.89	0.94	0.492	0.92	1.01

INSR	0.463	1.17	1.07	0.349	1.18	1.19
INSR	0.757	1.05	1.02	0.199	1.21	1.25
IPO11	0.097	1.29	1.26	0.585	1.12	1.26
IPO11	0.964	1.01	1.04	0.840	1.05	1.39
IPO9	0.629	0.91	1.06	0.487	0.87	1.09
IRF6	0.721	1.06	0.88	0.399	1.13	1.24
KRT20	0.795	1.16	1.09	0.670	0.78	0.26
LEMD1	0.147	0.83	0.95	0.736	0.97	1.00
LGALS2	0.359	0.75	1.07	0.798	0.93	1.28
LGALS3	0.938	0.99	1.05	0.675	0.93	0.93
LGALS3BP	0.472	1.10	1.08	0.238	1.17	1.16
LGALS3BP	0.286	1.20	1.04	0.175	1.42	1.21
LGALS8	0.054	0.31	0.30	0.679	0.78	0.83
LGLAS3BP	0.864	1.04	0.99	0.515	1.11	1.07
LINC00324	0.070	1.23	1.12	0.928	1.01	0.97
LINC00324	0.831	1.02	1.12	0.453	1.06	0.99
LOC130074	0.501	1.16	0.89	0.291	0.88	0.97
LOC130074	0.682	1.12	0.86	0.737	0.96	0.99
LOC142937	0.289	0.59	0.66	0.826	1.12	1.51
LPPR1	0.678	0.96	0.98	0.154	0.92	0.94
LPPR1	0.079	0.89	0.93	0.213	0.92	0.90
LRAT	0.223	0.91	0.89	0.868	0.99	0.98
LRAT	0.172	0.90	0.91	0.526	1.05	1.15
LTF	0.817	1.06	1.03	0.347	1.17	1.11
MAGEA2B	0.485	0.83	0.90	0.643	1.11	0.92
MALSU1	0.553	0.78	1.00	0.231	0.57	0.43
MAPK1	0.403	0.77	0.96	0.986	1.00	1.01
MAPK3	0.695	0.89	1.04	0.373	1.19	1.31
MGAT4B	0.762	0.91	1.08	0.527	0.85	0.84
MGAT4B	0.924	0.97	1.06	0.366	1.16	1.01
MGEA5	0.246	0.91	0.87	0.401	1.23	0.90
MGST1	0.755	0.97	0.93	0.375	0.94	1.03
MGST1	0.101	0.87	0.90	0.987	1.00	0.98
MNAT1	0.177	0.89	0.85	0.836	0.98	0.92
MPPE1	0.351	1.10	1.02	0.773	0.98	0.94
MPPE1	0.512	1.16	1.02	0.887	1.01	0.97
MPZL1	0.420	1.20	0.99	0.864	0.98	0.95
MPZL1	0.128	0.90	0.90	0.878	1.02	0.93
MRPL47	0.194	1.18	1.09	0.684	1.05	1.06
MRPL47	0.355	1.14	1.04	0.163	1.20	1.15
MRPS24	0.779	0.91	1.02	0.934	1.03	0.74
MRPS30	0.159	1.78	2.60	0.557	1.26	1.36

NNAT	0.294	1.26	0.97	0.942	1.01	0.99
OBFC2B/NABP2	0.512	0.91	0.84	0.104	1.24	0.96
PAGE2	0.520	1.21	1.11	0.655	0.94	0.94
PARD6A	0.933	0.99	0.96	0.374	1.16	1.10
PCLO	0.105	1.50	1.31	0.186	1.40	1.06
PGK1	0.800	1.09	0.91	0.586	1.14	1.04
PGM3	**0.004**	1.52	1.48	0.128	1.49	1.67
PHYHD1	0.744	0.95	1.09	0.828	0.98	0.93
PHYHD1	0.375	0.93	0.92	0.748	1.05	1.08
PKLR	0.266	0.82	0.80	0.432	1.18	1.19
PKM2	0.589	1.11	1.17	0.980	1.01	1.11
PKN1	0.121	0.68	0.87	0.470	1.20	1.20
PLA1A	0.084	0.87	0.96	0.941	0.99	1.02
PNOC	0.958	0.99	0.89	0.820	1.02	0.92
PNOC	0.148	0.88	0.92	0.867	1.02	0.96
POLR2L	0.902	1.02	0.86	0.740	1.04	1.06
PPBP	0.516	0.72	0.79	0.979	0.99	1.49
PPCDC	0.352	1.24	1.06	0.922	1.02	1.19
PRAME	0.442	1.09	1.17	0.287	1.32	1.07
PRAME	0.933	0.98	1.06	0.401	1.10	1.13
PRKAB2	0.638	0.92	1.06	0.979	1.00	1.11
PRKAB2	0.770	0.90	0.96	0.717	1.06	1.21
PRKD1	0.801	1.10	1.05	**0.024**	2.45	1.73
PSMA2	0.317	0.92	0.83	0.954	1.01	0.90
PSMB3	0.237	1.15	0.95	0.456	1.08	1.09
PSMB8	0.384	0.92	0.94	0.952	1.00	1.01
PSPH	0.554	0.79	0.81	0.968	1.01	0.91
RAB40B	0.111	0.86	0.82	0.821	0.98	0.86
RALBP1	0.492	0.85	1.19	0.752	1.07	1.03
RDH11	0.424	0.94	0.89	0.739	0.97	0.90
REPS1	0.887	0.98	0.99	0.844	1.02	0.82
REPS1	0.568	0.94	0.88	0.046	1.44	1.25
RLN1	0.397	0.62	1.00	0.777	1.13	1.00
RQCD1	0.082	1.28	1.23	0.652	0.95	0.94
RQCD1	0.115	0.85	0.90	0.245	1.32	1.08
SH3YL1	0.886	1.03	0.93	0.519	0.86	1.02
SLC35A2	0.467	0.94	0.91	0.374	1.09	1.04
SNF8	0.572	1.17	0.89	0.917	0.99	0.89
SPAG11B	0.115	0.89	0.85	0.788	0.97	0.98
SPHK1	0.748	1.11	1.36	0.374	1.31	1.31
STCH/HSPA13	0.089	0.86	0.83	0.460	1.12	1.10
STIP1	0.975	0.99	0.92	0.319	1.31	0.99

STK10	0.580	0.83	1.14	0.272	0.77	0.96
STK10	0.583	0.90	0.98	0.503	1.20	1.10
STK10	0.965	0.99	0.96	0.612	1.16	1.12
TADA3	0.662	1.07	0.92	0.576	0.93	0.79
TARP	0.575	0.95	0.92	0.947	0.99	0.88
TARP	0.220	0.87	0.86	0.869	1.02	0.99
TBCC	0.968	1.00	0.98	0.989	1.00	0.83
THAP4	0.884	1.01	1.09	0.870	0.98	0.89
TIMM44	0.984	0.99	0.96	0.993	1.00	0.94
TIMM44	0.239	0.77	0.93	0.812	1.04	1.12
TIMP1	0.332	1.31	1.27	0.869	0.99	1.02
TIMP1	0.246	0.94	0.93	0.748	1.06	1.06
TINAGL1	0.718	1.13	1.03	0.785	0.98	0.91
TINAGL1	0.533	0.95	0.93	0.955	1.01	1.02
TMC6	0.512	0.89	1.17	0.414	0.87	0.83
TMEM101	0.989	1.00	0.96	0.595	0.96	0.95
TMEM107	0.968	1.01	1.28	0.457	1.23	1.21
TMEM120A	0.570	0.97	0.93	0.660	0.96	0.96
TMEM43	0.820	1.06	1.07	0.817	0.97	0.93
TMEM43	0.098	0.66	0.83	0.408	1.26	1.10
TNPO3	0.189	0.92	0.93	0.605	0.96	0.82
TNPO3	0.165	0.67	0.79	0.673	0.95	0.89
TPI1	0.257	0.89	0.81	0.611	0.95	0.86
TRAF6	0.243	0.87	0.86	0.855	1.02	0.95
TRAF6	0.148	0.87	0.89	0.307	1.31	0.96
TRIM10	0.362	0.75	1.11	0.680	1.12	1.14
TRIM10	0.520	0.86	0.95	0.798	0.94	1.19
TRIML1	0.145	0.90	0.95	0.743	0.97	0.96
TTN	**0.005**	1.96	1.41	0.129	1.38	1.79
TXNDC14	0.449	0.94	0.88	0.935	0.99	0.98
TXNDC14	0.321	0.92	0.92	0.855	1.02	1.12
UBE2B	0.600	0.96	0.89	0.912	0.99	0.96
UCHL1	0.970	1.01	0.89	**0.046**	2.28	1.33
USP14	0.209	0.89	0.89	0.491	0.93	0.91
USP48	0.798	1.02	1.04	0.253	0.92	0.94
VANGL2	0.957	1.01	0.89	0.906	0.99	0.86
VANGL2	0.391	0.90	0.76	0.369	1.25	0.94
WTAP	0.252	0.70	0.90	0.473	1.21	1.28
XPO5	0.688	1.11	0.95	0.717	0.97	0.96
XPO5	0.096	0.89	0.88	0.840	0.98	0.92
XPOT	0.487	0.96	0.99	0.749	0.97	0.95
XPOT	0.923	0.99	0.89	0.906	1.01	1.08

XPOT	0.099	0.86	0.86		0.348	1.08	1.01
ZFAND3	0.388	0.79	1.04		0.263	0.77	0.66
ZG16	0.842	1.06	1.00		0.969	1.00	1.05
ZG16	0.213	0.74	0.88		0.948	1.01	0.97
ZNRF4	0.202	0.89	0.75		0.914	0.99	0.88
ZNRF4	0.119	0.89	0.85		0.321	1.26	1.06
IgG_1	0.915	1.05	2.20		0.876	0.98	0.98
IgG_1	0.301	0.87	0.82		0.380	1.47	1.74
IgG_2	0.507	1.30	1.00		0.744	1.04	1.04
IgG_2	0.220	0.86	0.93		0.244	1.59	1.00
EBNA_A	0.549	0.82	1.19		0.540	1.23	1.63
EBNA_B	0.843	0.95	0.96		0.217	1.36	1.19

ANA, Antinuclear Antibodies; EBNA, Epstein Barr Virus Nuclear Antigen 1

Case = women who later developed breast cancer, and Control = women who did not

[1]Unadjusted p-value for t-test comparing mean ratio Log2TAA to the null (1.0); shown in bold if Bold = p <0.05. Excludes Log2TAA values of zero. Total anti-TTA with one or more zero values = 71 breast cancer cases (26 ANA-positive, 45 ANA-negative), and 98 non-cases (46 ANA-positive, 52 ANA-negative); lowest count difference was not statistically significant (chi-square p=0.24).

[2]Median ratio of Log2TAA antibody levels: ANA-positive versus ANA-negative in cases and controls.

Supplemental Table 3. Correlations between EBNA and Log2 TAA reactivities from Table 2, stratified by ANA and case status.

			**Person Correlation Coefficients[1]**								
Antigen	Mean (SD)	Range	**EBNA**	**PGM3**	**TTN**	**DUSP26**	**PRKD1**	**UCHL1**	**ACTA2**	**C5orf45**	**C13orf24**
**ANA-positive breast cancer cases**											
EBNA	13.9 (1.7)	10-17	---	0.12	0.16	0.11	-0.13	-0.12	0.15	0.01	0.16
PGM3	8.5 (0.86)	6.7-10	---	---	0.07	0.26	0.05	0.06	**-0.40**	**-0.58**	-0.01
TTN	8.5 (1.4)	6.0-13	---	---	---	0.31	0.30	0.27	0.01	-0.17	0.02
DUSP26	9.9 (0.88)	7.3-12	---	---	---	---	-0.11	0.13	-0.15	-0.14	-0.19
PRKD1	4.5 (2.3)	0-7.7	---	---	---	---	---	0.30	-0.18	-0.05	0.02
UCHL1	9.4 (1.2)	6.2-13	---	---	---	---	---	---	**-0.41**	-0.09	**-0.55**
ACTA2	12 (0.5)	11-13	---	---	---	---	---	---	---	**0.59**	**0.41**
C5orf45	11 (0.7)	9.9-15	---	---	---	---	---	---	---	---	**0.39**
C13orf45	11 (0.41)	10-12	---	---	---	---	---	---	---	---	---
**ANA-negative breast cancer cases**											
EBNA	14.1 (1.7)	11-18	---	-0.13	0.01	-0.05	-0.26	-0.03	**0.59**	0.34	0.13
PGM3	8.2 (0.7)	6.8-10	---	---	-0.01	0.15	-0.09	0.20	-0.19	-0.22	-0.08
TTN	8.0 (1.1)	6.0-11	---	---	---	0.14	0.23	0.27	-0.20	0.07	0.03
DUSP26	9.6 (1.0)	7.3-11	---	---	---	---	-0.26	0.11	-0.29	**-0.62**	**-0.26**
PRKD1	4.4 (2.4)	0-7.7	---	---	---	---	---	0.29	-0.22	0.01	-0.04
UCHL1	9.4 (1.3)	6.2-13	---	---	---	---	---	---	-0.02	-0.10	-0.11
ACTA2	12 (0.4)	11-13	---	---	---	---	---	---	---	**0.62**	**0.39**
C5orf45	11 (0.8)	10-15	---	---	---	---	---	---	---	---	0.30
C13orf45	11 (0.7)	10-13	---	---	---	---	---	---	---	---	---
**ANA-positive controls**											
EBNA	13.9 (1.7)	11-17	---	-0.21	0.03	-0.10	-0.29	-0.18	0.04	0.04	0.15
PGM3	8.6 (1.5)	5.0-12	---	---	0.23	0.05	-0.04	0.30	0.06	0.18	0.18
TTN	8.1 (1.2)	5.3-12	---	---	---	-0.26	0.15	0.13	0.13	0.34	0.09

DUSP26	9.7 (1.1)	7.4-11	---	---	---	---	-0.32	0.04	-0.18	**-0.48**	-0.02
PRKD1	4.5 (2.5)	0-8.1	---	---	---	---	---	0.31	0.03	0.17	0.05
UCHL1	9.5 (2.4)	0-15	---	---	---	---	---	---	-0.15	0.04	0.17
ACTA2	12 (0.5)	10-13	---	---	---	---	---	---	---	**0.86**	**0.35**
C5orf45	11 (0.6)	9.7-12	---	---	---	---	---	---	---	---	0.31
C13orf45	11 (0.6)	10-14	---	---	---	---	---	---	---	---	---
**ANA-negative controls**											
EBNA	13.7 (1.8)	11-16	---	-0.19	0.12	0.05	-0.34	-0.19	0.34	0.05	0.29
PGM3	8.3 (1.7)	5.0-12	---	---	0.33	0.23	-0.04	0.25	**-0.53**	**-0.47**	**-0.43**
TTN	7.9 (1.3)	5.3-12	---	---	---	0.19	-0.16	0.06	-0.29	-0.11	-0.33
DUSP26	9.7 (1.2)	7.4-11	---	---	---	---	-0.30	-0.10	-0.25	**-0.39**	-0.20
PRKD1	3.9 (2.6)	0-7.2	---	---	---	---	---	0.35	0.00	0.17	0.10
UCHL1	8.9 (2.8)	0-13	---	---	---	---	---	---	-0.29	0.04	-0.31
ACTA2	12 (0.6)	10-13	---	---	---	---	---	---	---	**0.72**	**0.74**
C5orf45	11 (0.6)	9.7-12	---	---	---	---	---	---	---	---	**0.49**
C13orf45	11 (0.5)	9.7-12	---	---	---	---	---	---	---	---	---

ANA, Antinuclear Antibodies; EBNA, Epstein Barr Virus Nuclear Antigen 1

Case = women who later developed breast cancer, and Control = women who did not

[1]Coefficients shown in bold if $p < 0.05$

Supplemental Table 4. Mean Log2TAA values among cases, stratified by time to diagnosis and ANA positivity

		**Pre-clinical breast cancer cases**	
Anti-TAA		**7+ years to diagnosis**<br>(N=34)<br>Mean Log2TAA (SE)<br>ANA+/ANA-	**<7 years to diagnosis**<br>(N=35)<br>Mean Log2TAA (SE)<br>ANA+/ANA-
PGM3		8.5 (0.67) / 8.0 (0.63)	9.0 (1.0) / 8.3 (0.78)
TTN		8.7 (1.3) / 8.4 (1.1)	9.2 (1.8) / 7.7 (0.94)
DUSP26		10.0 (0.52) / 9.2 (1.3)	10.2 (0.71) / 10.0 (0.57)
PRKD1		4.6 (2.1) / 5.5 (1.5)	4.5 (2.6) / 3.5 (2.6)
UCHL1		9.5 (1.0) / 9.4 (1.2)	9.3 (1.4) / 9.3 (1.4)
ACTA2		11.8 (0.50) / 12.1 (0.41)	11.9 (0.43) / 12.1 (0.46)
C5orf45		10.9 (0.41) / 11.2 (1.1)	10.8 (0.48) / 11.1 (0.45)
C13orf24		11.0 (0.35) / 11.4 (0.68)	11.1 (0.46) / 11.3 (0.66)

Supplemental Table 5. Linear regression models showing the covariate-adjusted difference in mean Log2TAA intensities associated with ANA-positivity among controls and cases, stratified by time to diagnosis with imputed Log2TAA values[a]

	Controls	Pre-clinical breast cancer cases	
Antigens	No breast cancer diagnosis	7+ years to diagnosis	<7 years to diagnosis
	N=64	N=30	N=35
	Beta[b] (P-value)	Beta[b] (P-value)	Beta[b] (P-value)
PRKD1	**1.14 (0.022)**	**-1.29 (0.044)**	0.71 (0.34)
UCHL1	**1.18 (0.029)**	ND	ND

ND – not done (no Log2TAA zero values for UCHL1 in cases)

[a]When Log2TAA=0, missing values were imputed as half the value of the lowest value>0. For UCHL1 (2 ANA- controls, Log2TAA_i=3), for PRKD1 (14 controls, Log2TAA_i=1.2; N=3 cases 7+ to diagnosis years Log2TAA_i=1.3; N=10 cases <7 years Log2TAA_i=1.5.

[b]Betas, calculated through linear regression, show the difference in means in Log2TAA intensities for ANA+ versus ANA- women, adjusting for age, EBNA antibody titers, RF positivity, and current use of hormone therapy. Positive Beta value indicates higher Log2TAA intensity among ANA+ versus women (i.e., greater than zero), whereas a negative Beta value indicates lower Log2TAA intensity among ANA+ women. Sample size reduced due to missing covariate data in 7 cases and 8 controls.

Supplemental Table 6. Linear regression models showing the covariate-adjusted difference in Log2TAA levels associated with ANA-positivity among controls and among cases, stratified by time to diagnosis: age-adjusted and sensitivity analyses[1]

Antigens	Controls<br>No breast cancer diagnosis<br>(N=64)<br>Beta (P-value)	Pre-clinical breast cancer cases<br>7+ years to diagnosis<br>(N=30)<br>Beta (P-value)	<7 years to diagnosis<br>(N=35)<br>Beta (P-value)
PGM3 (Table 4)	0.64 (0.09)	0.42 (0.12)	0.56 (0.09)
Age-adjusted	0.58 (0.12)	0.53 (0.03)	0.64 (0.04)
Excl. lupus/DMARDS	0.61 (0.16)	0.54 (0.05)	0.48 (0.13)
Excl. prior cancer	NA	0.56 (0.03)	0.50 (0.11)
Limit to ER+ breast cancer	NA	0.60 (0.05)	0.64 (0.10)
TTN (Table 4)	0.44 (0.16)	0.11 (0.84)	1.63 (0.002)
Age-adjusted	0.47 (0.13)	0.31 (0.51)	1.54 (0.004)
Excl. lupus/DMARDS	0.32 (0.40)	0.31 (0.54)	1.50 (0.004)
Excl. prior cancer	NA	0.26 (0.59)	1.48 (0.006)
Limit to ER+ breast cancer	NA	0.17 (0.76)	1.21 (0.036)
DUSP26 (Table 4)	0.20 (0.49)	0.80 (0.07)	0.37 (0.12)
Age-adjusted	0.18 (0.50)	0.78 (0.04)	0.29 (0.20)
Excl. lupus/DMARDS	0.28 (0.38)	0.82 (0.04)	0.26 (0.30)
Excl. prior cancer	NA	0.78 (0.05)	0.31 (0.15)
Limit to ER+ breast cancer	NA	0.29 (0.58)	0.17 (0.56)
PRKD1[2]	1.36 (0.025)	-1.53 (0.044)	0.85 (0.38)
Age-adjusted	1.30 (0.02)	-0.90 (0.17)	1.08 (0.20)
Excl. lupus/DMARDS	1.24 (0.06)	-0.61 (0.37)	0.88 (0.32)
Excl. prior cancer	NA	-1.03 (0.13)	1.38 (0.11)
Limit to ER+ breast cancer	NA	-1.11 (0.19)	1.39 (0.21)
UCHL1	1.25 (0.032)	-0.25 (0.58)	-0.02 (0.97)
Age-adjusted	1.20 (0.04)	0.09 (0.82)	-0.05 (0.92)
Excl. lupus/DMARDS	0.92 (0.11)	0.18 (0.68)	0.17 (0.72)
Excl. prior cancer	NA	0.13 (0.75)	-0.01 (0.98)
Limit to ER+ breast cancer	NA		
ACTA2	-0.13 (0.28)	-0.13 (0.48)	-0.26 (0.041)

Age-adjusted	-0.06 (0.64)	-0.25 (0.13)	-0.24 (0.10)
Excl. lupus/DMARDS	0.02 (0.87)	-0.29 (0.10)	-0.22 (0.17)
Excl. prior cancer	NA	-0.26 (0.13)	-0.26 (0.09)
Limit to ER+ breast cancer	NA	-0.23 (0.25)	-0.16 (0.30)
C5orf45	-0.18 (0.15)	-0.52 (0.14)	-0.30 (0.031)
Age-adjusted	-0.11 (0.41)	-0.37 (0.19)	-0.30 (0.06)
Excl. lupus/DMARDS	-0.05 (0.71)	-0.36 (0.27)	-0.23 (0.15)
Excl. prior cancer	NA	-0.40 (0.18)	-0.27 (0.07)
Limit to ER+ breast cancer	NA	-0.41 (0.24)	-0.23 (0.17)
C13orf24	-0.01 (0.97)	-0.30 (0.14)	-0.32 (0.09)
Age-adjusted	0.00 (0.99)	-0.45 (0.02)	-0.26 (0.16)
Excl. lupus/DMARDS	0.05 (0.77)	-0.32 (0.05)	-0.30 (0.10)
Excl. prior cancer	NA	-0.46 (0.02)	-0.27 (0.17)
Limit to ER+ breast cancer	NA	-0.41 (0.24)	-0.23 (0.17)

DMARDS, disease modifying anti-rheumatic drugs; EBNA, Epstein Barr Virus Nuclear Antigen; ER+, estrogen-receptor positive

[1]Linear regression models calculated Beta values showing association with ANA), Values from Table 4 are shown for comparison, i.e., adjusted for EBNA, current hormone therapy, and rheumatoid factor positivity listed for comparison), and column heading indicates Ns from Table 4 adjusted models which varied due to missing covariate data contributing to Table 4 values. Therefore, the Ns for supplemental Table and sensitivity analyses are larger: N=72 controls; N= 34 longer time (7+ years) and N=39 shorter time (<7 years) to breast cancer diagnosis. Numbers also value depending on exclusion scenario: (1) Exclude lupus/DMARDs: N= 15 controls, N=3 longer time (7+ yrs) and N=4 shorter time (<7 yrs) to breast cancer diagnosis. (2) Exclude prior cancer: N=1 longer time (7+ yrs) and N=2 shorter time (<7 yrs) to breast cancer diagnosis. (3) Limit to ER-positive breast cancer: N=27 longer time (7+ yrs) and N=27 shorter time (<7 yrs) to breast cancer

[2]Substantial group confounding was seen among cases (i.e., adjusted for EBNA, current hormone therapy, and rheumatoid factor positivity). Example: ER+, <7 years to dx, adjusted beta=0.37 (0.78), decreased effect estimate; ER+, 7+ years to diagnosis, adjusted beta=-2.25 (0.01) increased effect estimate.
